# Supplementary material for: Quantifying coastal freshwater extremes during unprecedented rainfall using long timeseries multi-platform salinity observations
Source: Nat Commun. 2024 Jan 22;15:424. doi: 10.1038/s41467-023-44398-2 (PMC10803377; doi:10.1038/s41467-023-44398-2)
Supplement: Supplementary file 1 — Supplementary Information [file 41467_2023_44398_MOESM1_ESM.pdf]

Supplementary information for

Quantifying coastal freshwater extremes during unprecedented rainfall using long timeseries multi-platform salinity observations

Neil Malan*, Moninya Roughan, Michael Hemming, and Tim Ingleton

*Corresponding author. Email: n.malan@unsw.edu.au

1 Supplementary Text

Glider Quality Control and Processing

All IMOS gliders are equipped with a Seabird-CTD sensor, measuring temperature, salinity and depth, as well as a WETLabs ECO Puck measuring optical properties, such as coloured dissolved organic matter (CDOM). Glider data used in this study have undergone standard IMOS QA/QC procedures [3], and we apply further QC to the profiles, which are vertically gridded onto a 1 m grid. Temperature, salinity, and chlorophyll-a fluorescence are despiked using the median filter and standard deviation bounds methods from GliderTools [1]. The buoyancy frequency squared (N^2) and spice (the variation of temperature and salinity along a constant density surface) are calculated from absolute salinity and conservative temperature using the Gibbs seawater toolbox [2].

2 Supplementary Figure

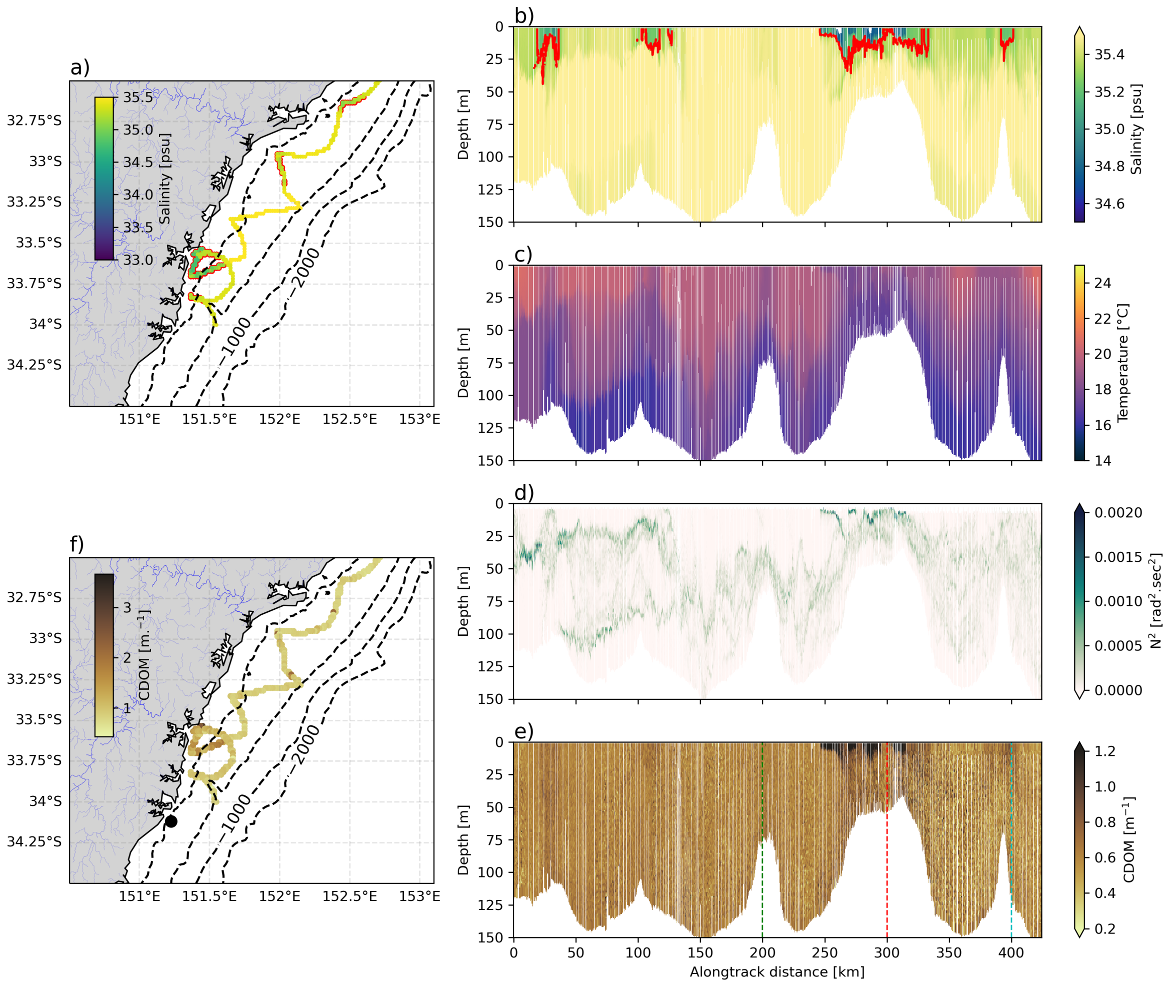

Figure S1: Case study of October 2022 glider mission. a) Glider track with minimum salinity for each profile (colours), and red outline for profiles were extreme low salinity water is present. Along-track sections for Salinity (b), Temperature (c), N^2 (d) and CDOM (e). f) Shows the glider track coloured by the maximum CDOM value for each profile.

.

3 Supplementary References

References

[1] Gregor, L., Ryan-Keogh, T. J., Nicholson, S. A., du Plessis, M., Giddy, I., and Swart, S. (2019). GliderTools: A Python Toolbox for Processing Underwater Glider Data. *Frontiers in Marine Science*, 6(December):1–13.

[2] McDougall, Trevor J. ; Barker, P. (2011). Getting started with TEOS-10 and the Gibbs Seawater (GSW) Oceanographic Toolbox. Technical report.

[3] Woo, M. (2019). Ocean Gliders delayed mode QA/QC best practice manual Version 2.0. Technical report.
